# Greater maintenance of bone mineral content in male than female athletes and in sprinting and jumping than endurance athletes: a longitudinal study of bone strength in elite masters athletes

**DOI:** 10.1007/s11657-020-00757-w

**Published:** 2020-06-10

**Authors:** Alex Ireland, Uwe Mittag, Hans Degens, Dieter Felsenberg, José L. Ferretti, Ari Heinonen, Erika Koltai, Marko T. Korhonen, Jamie S. McPhee, Igor Mekjavic, Jessica Piasecki, Rado Pisot, Zsolt Radak, Bostjan Simunic, Harri Suominen, Désirée C. Wilks, Keith Winwood, Jörn Rittweger

**Affiliations:** 1 Department of Life Sciences, Musculoskeletal Science and Sports Medicine Research Centre, Manchester Metropolitan University, John Dalton Building, Chester Street, Manchester, M1 5GD UK; 2 Institute of Aerospace Medicine, German Aerospace Center (DLR), Cologne, Germany; 3 Lithuanian Sports University, Kaunas, Lithuania; 4 University of Medicine and Pharmacy of Targu Mures, Targu Mures, Romania; 5 Osteology and Orphane Bone Diseases and Charité – Campus Benjamin Franklin, Centre of Muscle and Bone Research, Humboldt-University Berlin and Free University, Berlin, Germany; 6 Center for P-Ca Metabolism Studies (CEMFoC), National University of Rosario, Rosario, Argentina; 7 Faculty of Sport and Health Sciences, University of Jyväskylä, Jyvaskyla, Finland; 8 Research Institute of Sport Science, University of Physical Education, Budapest, Hungary; 9 Gerontology Research Center, Faculty of Sport and Health Sciences, University of Jyväskylä, Jyväskylä, Finland; 10 Department of Sport and Exercise Sciences, Musculoskeletal Science and Sports Medicine Research Centre, Manchester Metropolitan University, Manchester, UK; 11 Department of Automation, Biocybernetics and Robotics, Jozef Stefan Institute, Ljubljana, Slovenia; 12 Department of Biomedical Physiology and Kinesiology, Simon Fraser University, Burnaby, BC Canada; 13 Sport Health and Performance Enhancement Centre, Nottingham Trent University, Nottingham, UK; 14 Science and Research Centre Koper, Institute for Kinesiology Research, Koper, Slovenia; 15 Department of Pediatrics and Adolescent Medicine, University of Cologne, Cologne, Germany

**Keywords:** Exercise, Ageing, Biomechanics, Bone geometry, Osteoporosis

## Abstract

***Summary*:**

We investigated longitudinal changes in tibia bone strength in master power (jumping and sprinting) and endurance (distance) athletes of both sexes. Bone mass but not cross-sectional moment of inertia was better maintained in power than endurance athletes over time, particularly in men and independent of changes in performance.

**Objective:**

Assessment of effects of sex and athletic discipline (lower limb power events, e.g. sprint running and jumping versus endurance running events) on longitudinal changes in bone strength in masters athletes.

**Methods:**

We examined tibia and fibula bone properties at distal (4% distal-proximal tibia length) and proximal (66% length) sites using peripheral quantitative computed tomography (pQCT) in seventy-one track and field masters athletes (30 male, 41 female, age at baseline 57.0 ± 12.2 years) in a longitudinal cohort study that included at least two testing sessions over a mean period of 4.2 ± 3.1 years. Effects of time, as well as time × sex and time × discipline interactions on bone parameters and calf muscle cross-sectional area (CSA), were examined.

**Results:**

Effects of time were sex and discipline-dependent, even following adjustment for enrolment age, sex and changes in muscle CSA and athletic performance. Male sex and participation in power events was associated with better maintenance of tibia bone mineral content (BMC, an indicator of bone compressive strength) at 4% and 66% sites. In contrast, there was no strong evidence of sex or discipline effects on cross-sectional moment of inertia (CSMI, an indicator of bone bending and torsional strength—*P* > 0.3 for interactions). Similar sex and discipline-specific changes were also observed in the fibula.

**Conclusions:**

Results suggest that male athletes and those participating in lower limb power-based rather than endurance-based disciplines have better maintenance of bone compressive but not bending and torsional strength.

**Electronic supplementary material:**

The online version of this article (10.1007/s11657-020-00757-w) contains supplementary material, which is available to authorized users.

## Introduction

Bone mass decreases with age [1], contributing to increased fracture risk in older age [2]. An important contributor to this is reduced physical activity in middle and older ages [3, 4], particularly vigorous activities known to be osteogenic [5, 6]. Masters athletes continue to train and compete in athletics and thereby perform high levels of vigorous activity in old age. Cross-sectional studies show that they have greater bone mass, size and strength than less active age-matched peers [7–12]. These advantages are greater in lower limb power athletes (jumpers and sprinters) than endurance athletes [8–10, 12], but there are conflicting findings with regard to sex-specific effects of exercise on bone [8, 10, 12]. Cross-sectional studies also suggest that advantages in bone strength decline uniformly in both sexes and across disciplines over time despite continued regular participation in masters athletics [13, 14]. However, no studies have prospectively examined bone changes in masters athletes of different athletic disciplines over time. Therefore, we examined longitudinal changes in tibia and fibula bone outcomes in a cohort of international-standard power and endurance athletes of both sexes using peripheral quantitative computed tomography (pQCT). Primarily, we examined changes in bone mineral content (BMC, an indicator of bone compressive strength) and cross-sectional moment of inertia (CSMI, an indicator of bone bending and torsional strength). In particular, we assessed whether changes in bone strength were sex and/or discipline-specific.

## Methods

Eighty-six athletes in total were scanned at multiple timepoints, but fifteen were removed from analysis due to missing data. Therefore, seventy-one track and field masters athletes (aged 37–85 years) were included in this study (Table 1). Competing athletes were recruited at World, European and British Masters Athletic Championships between 2004 and 2014 (Supplementary Table 1). Recruitment targeted athletes who ranked highest in previous competitions or who had qualified for semi-finals or finals at the current event, and their mean age-graded performance was 85 ± 8% (where > 80% indicates national-class performance and > 90% world-class performance). Athletes were classified into two different event categories, depending on their self-rated best discipline as power (100/200/400 m, long/high/triple jump, pole vault) or endurance (800 m to marathon) athletes. Self-rated best discipline has previously been shown to be a valid indicator of individual athletic specialisation, showing 95% concordance with age-graded performances [12]. These athletes typically focus on one event, with the exception that one sprinter occasionally competed at 800 m and two distance runners occasionally competed in 400 m or sprint relay. With the exception of three pole vaulters, all jumpers also competed in sprint running but not distance running events. Baseline measures were taken between 2004 and 2008, with follow-up measures taken between 2005 and 2014.

**Table 1:** Cohort characteristics, separated by sex and discipline. Follow-up data indicate values at the final timepoint at which data were collected for each individual. †Complete data only available in 48 individuals (13 male power, 11 male endurance, 14 female power, 10 female endurance). AGP—age-graded performance, HRT—hormone replacement therapy

Variable		Baseline								Follow-up							
Sex		Males				Females				Males				Females			
Discipline		Power		Endurance		Power		Endurance		Power		Endurance		Power		Endurance	
n		16		17		24		14									
		Mean	SD	Mean	SD	Mean	SD	Mean	SD	Mean	SD	Mean	SD	Mean	SD	Mean	SD
Age (years)		65.1	13	60.2	11.9	53.5	10.7	54	10.5	68.8	13.4	65.1	11.8	57.2	11.8	58.7	11.9
Number of observations	2	13		10		22		11									
	3	2		3		0		4									
	4	1		1		1		1									
	5	0		0		1		1									
Observation time (years)		3.7	2.6	5	3.3	3.7	2.7	4.7	3.6								
Tibia length (mm)		384	24	378	17	358	17	354	23								
Muscle CSA (mm^2^)		8522	1041	7970	783	7222	761	6943	890	8531	905	7758	782	7164	737	6889	812
AGP (%)		89	5.7	83.5	9.1	85.1	7.4	83.9	8	89.5	8.5	81	8.4	87.4	8.9	86.2	7.7
Training volume (h/week)†		6.8	1.9	9.6	5.7	7.6	3.2	9.6	4.7	6.5	2.4	6.7	2.8	7.3	3	7.3	3.2
Postmenopausal (yes/no)						13/11		10/4						14/10		10/4	
Age at menopause (years)						52.6	2.4	46.8	7.1					52.1	3.1	46.8	7.1
HRT use (yes/no)	Current					4		0						4		0	
	Years of use					6	3.4	–						12	1.4	–	
	Previous					2		2						2		2	
	Years of use					9.6	13.3	4.3	5.3					9.6	13.3	4.3	5.3
	Never					18		12						18		12	

Exclusion criteria were pregnancy and musculoskeletal disorders known to affect the bones. Participants gave written informed consent before inclusion into the study, which had been approved by the Manchester Metropolitan University Department of Exercise and Sport Sciences Ethics Committee (approval number 2003/12/08). The British, European and World Master Athletics associations have been continuously involved with the design of the study. This was accomplished by discussions with both the associations and the athletes themselves and by providing feedback and inviting comment on completed studies.

Questionnaires were completed to assess sex and age. At each testing session, athletes were also asked to record the number of training hours per week, and women were asked for their menopausal state, age at menopause (if appropriate) and current and previous use of hormone-replacement therapy. Athletic performances (best time or jump/vault in the self-selected best event) during each championship were age-graded using the World Masters Athletes age-grading factors and the Age-Graded Performance (AGP) calculator available at http://www.howardgrubb.co.uk/athletics/wmalookup15.html, which expresses performances as a percentage relative to the age-specific world record.

Tomographic scans of the tibia and fibula were obtained with two XCT2000 and one XCT3000 scanners (STRATEC Medizintechnik GmbH, Pforzheim, Germany). Images were analysed with the integrated software version 6.00D. Cross-calibration was performed using the European Forearm Phantom, and images from the XCT2000 scanners were recalibrated according to derived equations, which all had coefficients of variation (*R*^2^) above 99.99% indicating near-perfect agreement. During the study, each scanner underwent daily quality assessment with a manufacturer-supplied phantom. Scans were obtained from the right side, unless a right-limb fracture within the last 24 months was reported. Tibia length was defined as the distance between medial knee-joint cleft and medial malleolus. Cross-sectional images were obtained at 4% and 66% distal-to-proximal tibia length after a scout scan viewing the tibio-talar joint. A voxel size of 0.5 mm in the transverse plane and of 2.4 mm in the longitudinal axis was used. The epiphyseal and diaphyseal bone measures were determined with segmentation thresholds of 180 mg/cm^3^ and 650 mg/cm^3^, respectively. Only the inner 45% of bone was selected for analysis of trabecular bone at the 4% site, using contour mode 1 and peeling mode 1. In addition, a region of interest was manually drawn around the outside of the calf muscle, but within the subcutaneous tissue layer, at the 66% site, and a threshold of 35 mg/cm^3^ was used to segment muscle and bone. Tibia and fibula total bone areas were subtracted from the derived area to give gross muscle cross-sectional area (CSA).

From the 4% site, total bone mineral content (BMC), total bone CSA and trabecular bone mineral density (BMD) were recorded. From the 66% site, total BMC, total bone CSA, cortical bone CSA, cortical BMD, cortical thickness and polar CSMI (indicating bone stiffness in torsion) were recorded. In vivo precision of pQCT measurements of the laboratory is described elsewhere and ranges between 0.2 and 0.5% for tibial total BMC and total and cortical bone CSA and 1.7% for CSMI [15, 16].

Statistical analyses were performed using the R statistical environment (version 3.2.2, www.r-project.org). Baseline sex differences in cohort characteristics and bone and muscle outcomes were compared using one-way ANOVA. Associations between age and muscle CSA and AGP were assessed using single-factor linear regression. To examine whether bone and muscle outcomes changed with time, linear mixed-effect models were created with a particular bone/muscle outcome as dependent variable, time as fixed effect and participant as random effect. Inclusion of a random participant effect allowed us to account for data clustering caused by differences in number of observations and time between observations. To investigate effects of sex and athletic discipline on changes over time, time × sex and time × discipline interactions were examined. Interaction terms were removed where *P* > 0.2 on the basis of highest *P* value until minimal models were obtained. Where interactions were identified (*P* < 0.1 for interaction term), subsequent models were sex and/or discipline-stratified. Where a sex interaction only was identified, models were created for men and women separately, and where a discipline interaction was identified, models were created for power and endurance athletes separately. Where both sex and discipline interactions were observed, four separate models were created separated by sex and discipline. Model 1 was adjusted only for enrolment age, sex and discipline; model 2 was additionally adjusted for muscle CSA and AGP. Inclusion of quadratic terms was used to test for deviation from linearity, but there was no evidence of non-linearity. Assuming a medium effect size (partial *η*^2^ = 0.09) and strong correlation between repeated measures (*r* = 0.75) given the high precision of pQCT scanners, a sample size of 14 per group would give 80% power to assess within-between factor interactions.

## Results

Men and women were re-scanned at a mean 4.3 ± 3.0 and 4.1 ± 3.1 years after baseline, respectively; 80% of participants were scanned twice, with the remainder scanned between 3 and 5 times (Table 1). Men were older than women, with a longer tibia length and greater muscle CSA, whilst endurance athletes had a greater weekly training volume than sprint athletes. The number of observations, time between observations and AGP were all comparable between sexes and disciplines (all *P* > 0.05). There was no association between age and muscle CSA or AGP for either sex at baseline. Whilst one woman reached menopause during the follow-up period, there were no changes in HRT use between timepoints. AGP increased marginally in women (+ 1.6%) but decreased by a small amount in men (− 2.1%) (both *P* < 0.05) over the study period. Training volume decreased from 8.2 ± 4.0 h per week to 7.0 ± 2.8 h per week (*P* = 0.02), but there were no effects of sex or discipline, or time × sex or time × discipline interactions (all *P* > 0.3).

At baseline, the majority of bone outcomes at all sites were larger in men than women, with the exception of cortical BMD at the proximal tibia which did not differ significantly between sexes (*P* = 0.44; Table 2). Distal tibia total BMC and CSA and proximal tibia total BMC, cortical CSA, cortical thickness and CSMI were all greater in power than endurance athletes (all *P* < 0.05). Distal fibula total BMC and proximal fibula total BMC, total CSA, cortical CSA and CSMI were greater in men whilst distal fibula total BMC, and proximal fibula cortical thickness was greater in power than endurance athletes.

**Table 2:** Tibia bone characteristics at baseline and follow-up. BMC—bone mineral content, CSA—cross-sectional area, BMD—bone mineral density, CSMI—cross-sectional moment of inertia

Site	Variable	Baseline								Follow-up								Baseline group differences (*P*)	
		Male				Female				Male				Female					
		Power		Endurance		Power		Endurance		Power		Endurance		Power		Endurance		Sex	Discipline
		Mean	SD	Mean	SD	Mean	SD	Mean	SD	Mean	SD	Mean	SD	Mean	SD	Mean	SD		
4%	Total BMC (mg/mm)	488	44	427	59	373	35	324	45	497	49	419	61	366	35	315	44	< 0.001	< 0.001
	Total bone CSA (mm^2^)	1410	144	1321	128	1136	124	1163	118	1427	138	1325	141	1138	118	1171	116	< 0.001	0.470
	Trabecular BMD (mg/mm^3^)	279	34	266	41	265	36	228	37	283	39	261	44	262	40	218	34	0.006	0.004
66%	Total BMC (mg/mm)	534	57	499	47	431	49	386	66	544	63	491	56	425	45	377	71	< 0.001	0.003
	Total bone CSA (mm^2^)	710	55	680	79	591	66	561	97	728	59	676	73	594	72	567	102	< 0.001	0.104
	Cortical bone CSA (mm^2^)	444	53	406	38	356	38	315	48	446	56	404	47	352	36	311	57	< 0.001	< 0.001
	Cortical BMD (mg/mm^3^)	1125	48	1132	33	1118	46	1122	51	1134	36	1116	43	1111	48	1100	43	0.444	0.603
	Cortical thickness (mm)	5.85	0.73	5.41	0.63	5.13	0.76	4.55	0.56	5.80	0.85	5.42	0.79	5.05	0.79	4.48	0.81	< 0.001	0.002
	CSMI (mm^4^)	79,548	12,372	72,811	14,777	54,134	10,901	47,938	14,246	82,589	12,335	72,158	14,708	54,204	10,740	48,053	14,850	< 0.001	0.040

**Fig. 1:**
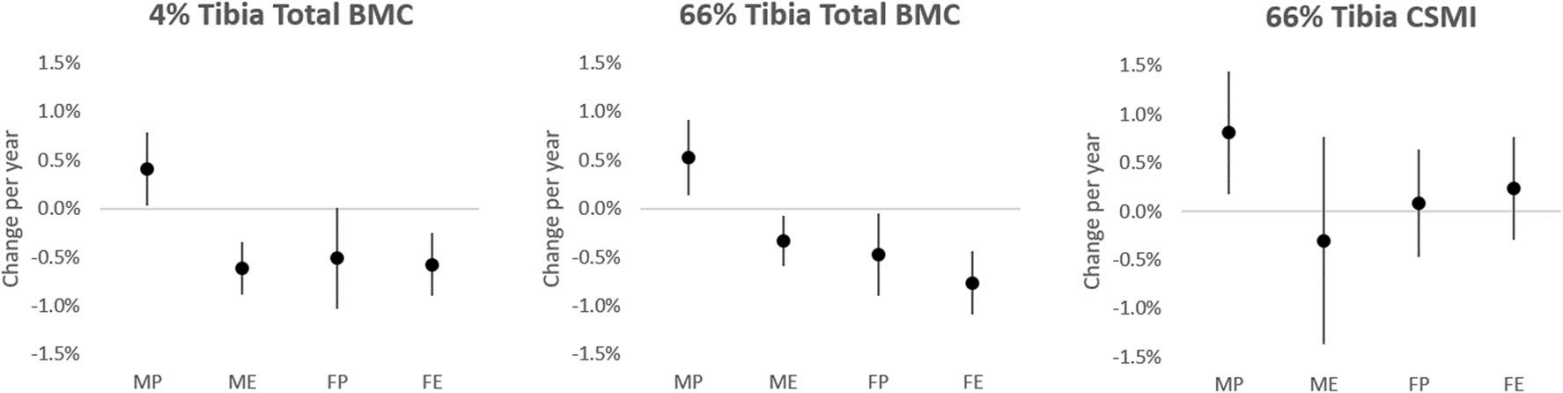
Percentage changes per year in primary tibia bone outcomes over the study period, calculated from regression coefficients following adjustment for enrolment age, sex, muscle CSA and AGP (model 2). Data points represent estimated mean value; error bars indicate 95% confidence intervals of the estimate. MP—male power, ME—male endurance, FP—female power, FE—female endurance

There was little effect of additional adjustment in model 2 in the majority of parameters (Table 3; Supplementary Table 3), therefore only the results of model 2 are presented. Based on model 2, we also calculated the percentage change per year for primary bone outcomes in the different groups (see Fig. 1). Tibia bone mineral content (indicating compressive bone strength) was better maintained in male and power athletes at both sites (Table 3; Fig. 1). At the trabecular-rich distal tibia site, this appears to result from better maintenance of trabecular BMD in these groups, whereas increases in total bone CSA were similar across sexes and disciplines (Supplementary Table 3). At the predominately cortical proximal tibia site, better maintenance of cortical BMD in males and sprint athletes and lower cortical thinning in male athletes appear to underly maintenance of total BMC in these groups. In contrast, there was no clear evidence of sex or discipline-specific changes in CSMI (indicating bending and torsional strength) which increased with time across the cohort as a whole. Muscle CSA decreased in men (change/year − 0.6%, 95% CI − 1.0 to − 0.2%) but was maintained in women (− 0.2%, 95% CI − 0.5 to 0.2%).

**Table 3:** Associations between time since initial observation and primary tibia bone outcomes. Where a time × sex or time × discipline interaction was observed (*P* < 0.1), analyses were performed separately for sex and/or discipline. Model 1 was adjusted for enrolment age, time, sex and discipline (except in sex/discipline-stratified analyses), and model 2 was additionally adjusted for muscle CSA and AGP. RC—regression coefficient, MP—male power, ME—male endurance, FP—female power, FE—female endurance, M—male, F—female

Site	Variable	Group	Model 1						Model 2					
			RC	95% CI		*P*	Time × sex	Time × disc	RC	95% CI		*P*	Time × sex	Time × disc
4%	Total BMC	MP	1.54	− 0.18	3.26	0.096	0.005	0.09	1.97	0.14	3.81	0.050	0.021	0.047
		ME	− 1.53	− 2.14	− 0.93	0.000			− 2.64	− 3.79	− 1.49	0.000		
		FP	− 2.42	− 3.99	− 0.85	0.005			− 1.91	− 3.83	0.01	0.062		
		FE	− 2.06	− 3.03	− 1.09	0.000			− 1.88	− 2.93	− 0.83	0.002		
66%	Total BMC	MP	2.87	0.73	5.01	0.017	0.001	0.005	2.83	0.43	5.22	0.033	0.002	0.008
		ME	− 1.65	− 2.92	− 0.38	0.020			− 2.76	− 4.97	− 0.56	0.026		
		FP	− 2.06	− 3.89	− 0.23	0.035			− 2.44	− 4.65	− 0.23	0.040		
		FE	− 2.95	− 4.20	− 1.70	0.000			− 3.03	− 4.44	− 1.61	0.000		
	CSMI		157	− 17	332	0.080	0.4	0.705	208	30	385	0.024	0.3	0.492

Distal and proximal fibula BMC bone losses were lower in male than female athletes (Supplementary Table 4). At proximal fibula, this appears to result from better maintenance of cortical CSA and BMD. Proximal BMC loss was lower in power than endurance athletes (Supplementary Table 4), as a result of better maintenance of cortical BMD. Decreases in proximal fibula CSMI were similar between sexes and disciplines, as were decreases in total bone CSA (Supplementary Table 4).

Training volume data at baseline and follow-up was available in 48 participants, with 69 participants having partial data. Training volume was not independently associated with any bone parameter, and additional adjustment of model 2 for training volume did not affect observed associations. In addition, further adjustment for menopause and HRT use in women did not substantially influence results. Rapid perimenopausal bone loss occurs from around 2–3 year pre-menopause until 3–4 years postmenopause [17], therefore in additional analyses we removed 9 perimenopausal women (6 power athletes, 3 endurance athletes) within 5 years of menopause at any timepoint. This resulted in attenuation of sex × time interactions for distal tibia BMC and BMD and distal fibula BMC (Supplementary Table 4), although sex interactions for the proximal tibia and fibula outcomes were still evident.

## Discussion

In this study, we observed sex- and discipline-specific changes; tibia bone compressive strength (indicated by BMC) was better maintained in male and power athletes. At the trabecular-rich distal tibia site, these differences were explained by better maintenance of trabecular BMD, whilst tibia bone CSA increased with time independently of sex and discipline at a greater rate than previously observed in non-athletes. At the cortical proximal site, better maintenance of BMC in male and power athletes resulted from absence of cortical BMD losses and a lower level of cortical thinning in males. In contrast, changes in tibia bending and compressive strength (indicated by CSMI) were similar in athletes of both sexes and disciplines. A similar pattern of sex- and discipline-specific changes occurred in the fibula, with the exception that total bone CSA and CSMI decreased similarly over time in all groups. Changes in bone traits were independent of changes in muscle CSA and athletic performance. These data suggest that bone mass is better preserved in male than female athletes and that power training is better for maintenance of bone mass than endurance training in older age.

### Comparison with previous findings

This is the first study to examine longitudinal changes in detailed bone characteristics of masters athletes using pQCT, which has greater precision and allows more detailed assessment of bone characteristics than DXA. Previous cross-sectional pQCT studies of masters athletes [13] and tennis players [14] found that bone strength advantages attributable to exercise were smaller in older than younger individuals. The virtual absence of tibial bone losses found for male sprinters in the present study was not picked up by these previous cross-sectional studies which suggested similar decreases in athletes regardless of sex or discipline. This re-emphasises the necessity of longitudinal studies before drawing conclusions based on cross-sectional data alone. The within-individual assessment of bone changes in this study increased our ability to detect sex and discipline-specific effects. Female athletes of both disciplines had greater rates of bone loss than men in agreement with longitudinal pQCT studies of non-athletic individuals [18]. The only other longitudinal studies on bone in masters athletes of both sexes suggest that bone loss was either absent in both sexes [19, 20] or was broadly similar [13]. Our study differed from those previously by the much wider age range covered, by inclusion of power athletes and by the use of pQCT, all of which explains why the apparently positive effect of sprinting and jumping has gone unnoticed so far. Our study agrees with the previous DXA-based study [19] in finding no effects of HRT on bone parameters in female athletes. Moreover, calf muscle CSA decreased in men but not in women, in contrast to findings of age-related declines in both sexes in a previous cross-sectional pQCT study of non-athletes [21].

**Fig. 2:**
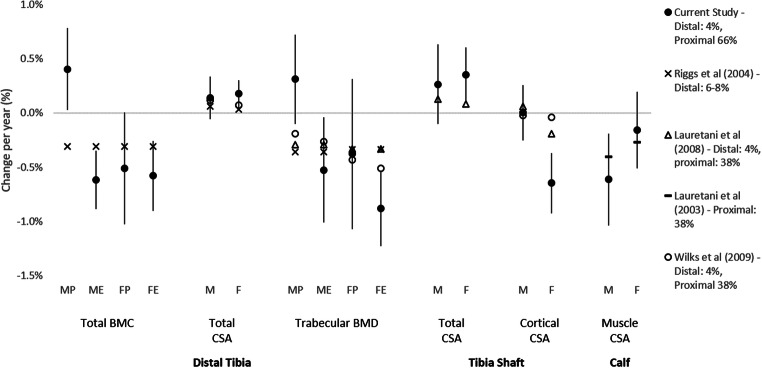
Comparison of age-related changes observed in this study with previous cross-sectional (Riggs et al. [1]) and longitudinal (Lauretani et al. [18] and Lauretani et al. [21]) studies in non-athletic individuals [1, 18, 21] and a cross-sectional study of masters athletes (Wilks et al. [13]). Figure legend includes study details and location of tibia sites examined

Although non-athletic controls were not recruited in this study, there are large cross-sectional [1] and longitudinal [18] population studies at hand that can be used. The most direct comparisons can be made with the work by Lauretani and colleagues [18]. They also examined proximal tibia properties at the 4% and 38% site (rather than 66% site used in this study), whilst Riggs and colleagues [1] examined bone at a fixed distance from tibia end equivalent to ~6–8% tibia length. In addition, in a previous cross-sectional masters athlete study, we examined associations between age and tibia bone outcomes at 4% and 38% tibia length using similar analyses. For women and male endurance runners, the age effects on bone observed within the present study are comparable with longitudinal studies by Lauretani et al. [18] and cross-sectional study by Riggs et al. [1], as well as our previous cross-sectional study (Fig. 2). However, preservation or possible increases with age in distal tibia total BMC and trabecular BMD were unique to male power athletes from the present study. In addition, trabecular BMD of male power athletes in the current study was better maintained than previously reported from cross-sectional studies, although rates of trabecular BMD decline in female sprinters and in endurance athletes of both sexes were similar. It is also noteworthy that rates of distal tibia total BMC loss in male endurance runners, trabecular BMD in female endurance runners and proximal tibia cortical CSA in females were higher than sex-matched values previously reported for non-athletic cohorts. In addition, the rates of total bone CSA increase at 4% and 66% sites in women were greater than those observed previously [1, 18].

### Potential mechanisms of findings

Female athletes had a markedly greater rate of bone mass decline than male athletes, when compared to differences observed in non-athletic individuals [18]. Given that most female athletes were postmenopausal this could relate to the effects of oestrogen on mechanical sensitivity of bone [22–24]. It has been shown that greater total bone CSA is associated with greater rates of bone mass loss [25]; therefore, rapid bone CSA increases in female athletes may contribute. These bone CSA increases may result from lower levels of oestrogen (known to suppress periosteal apposition [24]) in female athletes than non-athletes [26]. Female athletes consequently have larger bones than non-athletes even at unloaded sites [12, 27]; therefore, hormonal effects of exercise may influence longitudinal bone health changes. Only nine female athletes were within 5 years of menopause at any study timepoint, and removal of these individuals from analysis resulted in attenuation of sex interactions at distal but not proximal tibia and fibula. This suggests that sex-specific findings that we have reported in the shaft are not simply a result of changes relating to menopause.

Bone losses were greater in endurance than power athletes, despite a similar AGP and even greater training hours in the former. Endurance masters athletes complete more lower-impact physical activity than power athletes [8]. There is some evidence that this less vigorous activity has negative effects on bone strength [5, 8] potentially by reducing bone mechanosensitivity [28], which may explain discipline-specific effects observed in this study. In addition, declines in running speed are greater in endurance than sprint runners [29]; therefore, despite little change in AGP, mechanical loading may decrease more in endurance than sprint athletes with time. Alternatively, it has been proposed that calcium loss through excessive sweating during prolonged endurance exercise could contribute to bone loss [30]. Although nutrition is also an important determinant of exercise efficacy and may have contributed to discipline-specific findings in this study, this is unlikely. However, little is currently known about the diets of elite masters athletes in different disciplines [31] and this information was not collected in the current study. As the greatest stressor of bone, changes in muscular output could contribute to discipline and sex-specific patterns of bone change. Muscle CSA decreases were greater in men than women, but similar between disciplines, and additional adjustment for muscle CSA did not attenuate sex × time or discipline × time interactions for the age-related changes in the bone. Whilst muscle CSA is only an indicator of muscle output, previous studies of masters athletes found similar declines in muscle output (jumping power) in athletes of both sexes across athletic disciplines [32], and muscle power seems beneficial for bone strength [33, 34].

Relatively little is known about fibula changes with exercise and ageing. Fibula bone strength of power and endurance athletes appears similar or lower than controls [35–37], in contrast to large advantages observed in the tibia [12, 35]. The age-related decrease in fibula bone CSA is striking and contrary to increased size usually observed in long bones with age [18, 38]. However, the only previous cross-sectional study we are aware of which examined fibula ageing found lower fibula CSA in older individuals [35]. There is precedent for observations of decreasing long bone CSA, which occurs during development to maintain joint shape [39], and has also been observed in the tibia in longitudinal studies of the very old [18].

### Strengths and limitations

One of the strengths of this study was the relatively large number of highly-competitive masters athletes of both sexes who were examined longitudinally for changes in bone health. It would be difficult to motivate study participants of exercise intervention trials to train with the same vigour with which masters athletes train and compete. In that sense, our study provides a unique insight into the potential for osteogenic effects of exercise at older age. Assessment of AGP showed that participants maintained a high level of competitive performance throughout the study period and adjustment for AGP and reduced training volume did not explain study findings. Although there may have been a selection bias, we feel that the results of this study suggest that tibial bone mass is at least maintained in male masters power athletes, although losses were observed in females and endurance runners. Thus, sprint running and hopping, as a plyometric type of exercise, may offer bone benefits that cannot be achieved by endurance running. Notably, this positive effect was independent of muscle CSA. This observation in masters athletes is supported by the fact that plyometric exercises are particularly osteogenic in children [40] and that they are also a potent tool to prevent bed rest-induced bone loss [41]. Moreover, recent findings of a randomised controlled trial by Suominen et al suggest that sprint and strength training can improve bone health even in individuals already participating in high-impact exercise [42]. Participant age and length of observation varied substantially within our cohort, which may have influenced results. However, we found no evidence of non-linear changes in the bone with time and adjusted for group differences in enrolment age. In addition, our study design involved complex interactions of sex, age and athletic discipline which may be difficult to unpick, although the consistency and statistical strength of the sex and discipline interactions we observed across multiple bones and sites is reassuring. We grouped athletes by discipline using a binary classification (power/endurance) on the basis of self-selected best event, which we have shown previously to be a highly-valid predictor of performance [12]. This distinction has been shown to be sensitive enough to detect group differences in the bone at multiple sites [8]. Whilst many athletes compete in multiple events, detailed examination of event participation showed that the vast majority of power and endurance athletes only competed in one discipline of events. Any minimal overlap between the two is likely to impair our ability to detect group differences rather than introduce spurious findings. Related to this, we included both sprint and jump athletes in the power discipline which have different movements. However, our previous findings of similar bone strength between sprint and jump athletes [43] as well as muscle function (unpublished data in preparation) suggest that these events have similar effects on bone. In addition, almost all jumpers also participated in sprint running events whilst the three pole vaulters who did not participate already have a phase of sprinting as part of their event. As with any observational study, our findings may be influenced by more detailed training or other characteristics not measured and replication in an interventional model is required. This would also address the question of how bone changes in athletes relate to those in non-athletic individuals. Whilst we made some efforts to compare our results to those from previous similar cross-sectional and longitudinal studies, we recognise that the lack of a defined control group limits our ability to generalise our findings to a wider population. Finally, the inclusion of perimenopausal women may have influenced our results due to the substantial bone changes occurring at this time. Sensitivity analyses suggested that similar results were observed in non-perimenopausal women, but further studies investigating menopausal interactions with exercise and bone are justified.

## Conclusions

The question arises how the bone benefits of sprinting and jumping could become accessible to a wider population. Masters athletes continue to train at high intensity, which will not be appealing or even possible for all older adults. However, paradigms for old-age exercise already shifted from endurance-type to strength-type exercises a few decades ago [44], and the current trend seems to be going towards high-intensity interval training (HIIT) [45, 46]. Including plyometric exercises into HIIT prescriptions seems quite feasible, and it could be worthwhile if older people could thereby reap benefits for their bones. In conclusion, bone mass is better maintained in male than female masters athletes, whilst regular power-based training and competition in sprinting and jumping is associated with better maintenance of bone mass, but not bending or torsional strength, than endurance running.

## Electronic supplementary material

ESM 1 (DOCX 32 kb)

## Data Availability

Deidentified participant data are available upon reasonable request from the first author.
