# Supplementary material for: Greater maintenance of bone mineral content in male than female athletes and in sprinting and jumping than endurance athletes: a longitudinal study of bone strength in elite masters athletes
Source: Arch Osteoporos. 2020 Jun 10;15(1):87. doi: 10.1007/s11657-020-00757-w (PMC7286845; doi:10.1007/s11657-020-00757-w)
Supplement: Supplementary file 1 — (DOCX 32 kb) [file 11657_2020_757_MOESM1_ESM.docx]

Supplementary Table 1. Details of events at which athletes were recruited for this study

| Competition | Year | Location |
| --- | --- | --- |
| World Master Athletics Championships | 2005 | San Sebastian, Spain |
|  | 2009 | Lahti, Finland |
| European Masters Athletics Championships | 2004 | Aarhus, Denmark |
|  | 2006 | Poznań, Poland |
|  | 2008 | Ljubljana, Slovenia |
|  | 2010 | Nyíregyháza, Hungary |
| British Masters Athletic Championships | 2004 | Birmingham, UK |
|  | 2014 | Birmingham, UK |
| British Inter-Area Championships | 2014 | Birmingham, UK |

Supplementary Table 2. Fibula characteristics at baseline and follow-up.

| Bone | Site | Variable | Baseline | | | | | | | | Follow-up | | | | | | | |
| --- | --- | --- | --- | --- | --- | --- | --- | --- | --- | --- | --- | --- | --- | --- | --- | --- | --- | --- |
|  |  |  | Male | | | | Female | | | | Male | | | | Female | | | |
|  |  |  | Power | | Endurance | | Power | | Endurance | | Power | | Endurance | | Power | | Endurance | |
|  |  |  | Mean | SD | Mean | SD | Mean | SD | Mean | SD | Mean | SD | Mean | SD | Mean | SD | Mean | SD |
| Fibula | 4% | Total BMC(mg.mm^-1^) | 116 | 20 | 103 | 13 | 95 | 17 | 83 | 13 | 117 | 20 | 100 | 13 | 92 | 16 | 80 | 13 |
|  |  | Total Bone CSA (mm^2^) | 197 | 42 | 177 | 24 | 176 | 29 | 175 | 29 | 198 | 44 | 177 | 25 | 171 | 25 | 172 | 27 |
|  | 66% | Total BMC(mg.mm^-1^) | 100 | 17 | 96 | 12 | 89 | 16 | 82 | 16 | 99 | 18 | 92 | 15 | 85 | 16 | 76 | 17 |
|  |  | Total Bone CSA (mm^2^) | 111 | 22 | 107 | 14 | 91 | 15 | 88 | 14 | 108 | 18 | 105 | 12 | 90 | 14 | 89 | 14 |
|  |  | Cortical Bone CSA (mm^2^) | 86 | 17 | 82 | 11 | 76 | 13 | 70 | 12 | 84 | 17 | 80 | 13 | 74 | 13 | 66 | 14 |
|  |  | Cortical BMD (mg.mm^-3^) | 1106 | 53 | 1121 | 38 | 1117 | 46 | 1112 | 55 | 1113 | 45 | 1097 | 51 | 1104 | 46 | 1073 | 58 |
|  |  | Cortical Thickness (mm) | 3.15 | 0.52 | 3.06 | 0.47 | 3.30 | 0.61 | 2.91 | 0.45 | 3.14 | 0.59 | 3.01 | 0.59 | 3.16 | 0.62 | 2.67 | 0.63 |
|  |  | CSMI (mm^4^) | 2242 | 847 | 2100 | 572 | 1760 | 527 | 1592 | 483 | 2155 | 744 | 2058 | 585 | 1693 | 493 | 1556 | 506 |

Supplementary Table 3. Associations between time since initial observation and secondary tibia bone outcomes. Where a time*sex or time*discipline interaction was observed (P < 0.1), analyses were performed separately for sex and/or discipline. Model 1 was adjusted for age at enrolment, time, sex and discipline^†^, and Model 2 was additionally adjusted for muscle CSA and AGP (except for muscle CSA, where data were only adjusted for AGP). RC – regression coefficient, CI – confidence interval, MP – male power, ME – male endurance, FP – female power, FE – female endurance, M – male, F – female. ^†^Except in sex/discipline-stratified analyses.

| Bone | Site | Variable | Group | Model 1 | | | | | | | | | Model 2 | | | | | | |
| --- | --- | --- | --- | --- | --- | --- | --- | --- | --- | --- | --- | --- | --- | --- | --- | --- | --- | --- | --- |
|  |  |  |  | RC | 95%CI | | | P | | Time* Gender | Time* Disc | RC | | 95%CI | | P | Time* Gender | Time* Disc |  |
| Tibia | 4% | Total CSA | | 1.429 | 0.269 | 2.589 | 0.018 | | 0.909 | | 0.687 | 1.527 | | 0.313 | 2.741 | 0.016 | 0.925 | 0.613 |  |
|  |  | Trabecular BMD | MP | 0.780 | -0.254 | 1.813 | 0.156 | | 0.008 | | 0.008 | 0.872 | | -0.293 | 2.037 | 0.160 | 0.036 | 0.005 |  |
|  |  |  | ME | -1.255 | -1.879 | -0.631 | 0.001 | |  |  |  | -1.369 | | -2.634 | -0.105 | 0.050 |  |  |  |
|  |  |  | FP | -1.249 | -2.736 | 0.238 | 0.111 | |  |  |  | -1.002 | | -2.835 | 0.831 | 0.294 |  |  |  |
|  |  |  | FE | -2.342 | -3.112 | -1.573 | 0.000 | |  |  |  | -2.022 | | -2.818 | -1.225 | 0.000 |  |  |  |
|  | 66% | Total CSA | | 2.012 | 0.630 | 3.394 | 0.005 | | 0.616 | | 0.606 | 2.353 | | 0.959 | 3.746 | 0.001 | 0.781 | 0.774 |  |
|  |  | Cortical CSA | M | -0.123 | 0.921 | 0.795 | 0.801 | | 0.008 | | 0.119 | 0.016 | | -1.069 | 1.100 | 0.978 | 0.002 | 0.207 |  |
|  |  |  | F | -1.838 | -2.659 | -1.017 | 0.000 | |  |  |  | -2.187 | | -3.119 | -1.254 | 0.000 |  |  |  |
|  |  | Cortical BMD | MP | 2.649 | -0.483 | 5.782 | 0.114 | | 0.018 | | <0.001 | 1.543 | | -1.581 | 4.667 | 0.347 | 0.055 | <0.001 |  |
|  |  |  | ME | -3.544 | -5.429 | -1.659 | 0.002 | |  |  |  | -3.391 | | -5.799 | -0.984 | 0.014 |  |  |  |
|  |  |  | FP | -1.886 | -5.107 | 1.336 | 0.261 | |  |  |  | -1.273 | | -5.061 | 2.515 | 0.516 |  |  |  |
|  |  |  | FE | -6.043 | -8.223 | -3.863 | 0.000 | |  |  |  | -5.666 | | -7.970 | -3.362 | 0.000 |  |  |  |
|  |  | Cortical Thickness | M | -0.009 | -0.036 | 0.017 | 0.500 | | 0.062 | | 0.197 | -0.005 | | -0.034 | 0.024 | 0.740 | 0.02 | 0.317 |  |
|  |  |  | F | -0.041 | -0.063 | -0.020 | 0.000 | |  |  |  | -0.046 | | -0.069 | -0.022 | 0.000 |  |  |  |
| Calf Muscle CSA | | | M | -44.1 | -77.4 | -10.9 | 0.013 | | 0.070 | | 0.297 | -50.4 | | -85.3 | -15.6 | 0.007 | 0.053 | 0.344 |  |
|  |  |  | F | -8.4 | -30.6 | 13.8 | 0.462 | |  |  |  | -11.3 | | -36.3 | 13.8 | 0.382 |  |  |  |

Supplementary Table 4. Associations between time since initial observation and fibula bone outcomes. Where a time*sex or time*discipline interaction was observed (P < 0.1), analyses were performed separately for sex and/or discipline. Model 1 was adjusted for enrolment age, time, sex and discipline^†^, and Model 2 was additionally adjusted for muscle CSA and AGP (except for muscle CSA, where data were only adjusted for AGP). RC – regression coefficient, CI – confidence interval, MP – male power, ME – male endurance, FP – female power, FE – female endurance, M – male, F – female. ^†^Except in sex/discipline-stratified analyses.

| Site | Variable | Group | Model 1 | | | | | | Model 2 | | | | | |
| --- | --- | --- | --- | --- | --- | --- | --- | --- | --- | --- | --- | --- | --- | --- |
|  |  |  | RC | 95%CI | | P | Time* Gender | Time* Disc | RC | 95%CI | | P | Time* Gender | Time* Disc |
| 4% | Total BMC | M | -0.391 | -0.649 | -0.133 | 0.005 | 0.018 | 0.242 | -0.421 | -0.707 | -0.135 | 0.007 | 0.056 | 0.19 |
|  |  | F | -0.807 | -1.028 | -0.586 | 0.000 |  |  | -0.828 | -1.094 | -0.561 | 0.000 |  |  |
|  | Total CSA | | -0.625 | -0.917 | -0.333 | 0.000 | 0.475 | 0.454 | -0.587 | -0.886 | -0.287 | 0.000 | 0.407 | 0.442 |
| 66% | Total BMC | MP | -0.099 | -0.807 | 0.610 | 0.788 | 0.01 | 0.018 | 0.145 | -0.656 | 0.947 | 0.727 | 0.003 | 0.023 |
|  |  | ME | -1.002 | -1.508 | -0.495 | 0.001 |  |  | -0.937 | -1.714 | -0.159 | 0.031 |  |  |
|  |  | FP | -1.075 | -1.685 | -0.464 | 0.002 |  |  | -1.293 | -2.008 | -0.578 | 0.002 |  |  |
|  |  | FE | -1.569 | -1.970 | -1.169 | 0.000 |  |  | -1.595 | -2.047 | -1.143 | 0.000 |  |  |
|  | Total CSA | | -0.292 | -0.537 | -0.047 | 0.021 | 0.269 | 0.2 | -0.234 | -0.480 | 0.012 | 0.065 | 0.194 | 0.921 |
|  | Cortical CSA | M | -0.455 | -0.814 | -0.096 | 0.018 | 0.019 | 0.224 | -0.266 | -0.683 | 0.151 | 0.220 | 0.001 | 0.277 |
|  |  | F | -0.994 | -1.268 | -0.720 | 0.000 |  |  | -1.101 | -1.407 | -0.795 | 0.000 |  |  |
|  | Cortical BMD | MP | 1.805 | -2.074 | 5.684 | 0.373 | 0.005 | <0.001 | 0.416 | -3.213 | 4.045 | 0.825 | 0.02 | <0.001 |
|  |  | ME | -5.382 | -7.614 | -3.150 | 0.000 |  |  | -5.014 | -7.727 | -2.301 | 0.002 |  |  |
|  |  | FP | -3.246 | -6.396 | -0.096 | 0.053 |  |  | -2.975 | -6.690 | 0.740 | 0.129 |  |  |
|  |  | FE | -8.908 | -11.090 | -6.725 | 0.000 |  |  | -8.299 | -10.49 | -6.109 | 0.000 |  |  |
|  | Cortical Thickness | M | -0.010 | -0.024 | 0.005 | 0.198 | <0.001 | 0.008 | -0.009 | -0.024 | 0.007 | 0.284 | <0.001 | 0.124 |
|  |  | F | -0.051 | -0.065 | -0.038 | 0.000 |  |  | -0.054 | -0.070 | -0.039 | 0.000 |  |  |
|  | CSMI | | -16.1 | -24.8 | -7.4 | 0.000 | 0.479 | 0.712 | -15.0 | -24.0 | -6.0 | 0.002 | 0.734 | 0.703 |
